# Supplementary material for: Understanding older adults’ adoption of facial recognition payment: An integrated model of TAM and UXT
Source: PLoS One. 2025 Jul 10;20(7):e0325291. doi: 10.1371/journal.pone.0325291 (PMC12244727; doi:10.1371/journal.pone.0325291)
Supplement: Attachment — Submitted filename: Response to Reviewers .docx [file pone.0325291.s003.docx]

**Response to Reports on Manuscript entitled “Are Facial Recognition Payments Aging-Friendly? Investigating Acceptance Through an Extended Technology Acceptance Model”**

**(No.: PONE-D-24-56273).**

We sincerely appreciate the valuable feedback provided by Editor Simon Dang. Below are our detailed responses to the journal requirements and the reviewers’ comments. For clarity, the journal requirements are presented in a tabular format, while the editor’s feedback is displayed in paragraph form. Additionally, the journal requirements and the editor’s comments are highlighted in light blue italics, whereas our responses are presented in black non-italicized text. In addition to the revisions made in response to the editor’s feedback, we have also made adjustments to other parts of the manuscript. For details regarding these modifications, please refer to the instructions at the end of the manuscript, where the title is highlighted in red.

I. ***Journal Requirements:***

| *Question* | *Response* |
| --- | --- |
| *1. Please ensure that your manuscript meets PLOS ONE's style requirements, including those for file naming. The PLOS ONE style templates can be found at https://journals.plos.org/plosone/s/file?id=wjVg/PLOSOne_formatting_sample_main_body.pdf and https://journals.plos.org/plosone/s/file?id=ba62/PLOSOne_formatting_sample_title_authors_affiliations.pdf* | We have accessed the journal's formatting templates provided in the links and converted the PDF files into Word format. Following the journal’s formatting requirements, we have meticulously reformatted the manuscript, including the main text, title, author names, and affiliations, to ensure full compliance with the prescribed guidelines. |
| *2. PLOS requires an ORCID iD for the corresponding author in Editorial Manager on papers submitted after December 6th, 2016. Please ensure that you have an ORCID iD and that it is validated in Editorial Manager. To do this, go to ‘Update my Information’ (in the upper left-hand corner of the main menu), and click on the Fetch/Validate link next to the ORCID field. This will take you to the ORCID site and allow you to create a new iD or authenticate a pre-existing iD in Editorial Manager.* | We have included the corresponding author’s ORCID iD in the submission system. |
| *3. Please provide additional details regarding participant consent. In the ethics statement in the Methods and online submission information, please ensure that you have specified what type you obtained (for instance, written or verbal, and if verbal, how it was documented and witnessed). If your study included minors, state whether you obtained consent from parents or guardians. If the need for consent was waived by the ethics committee, please include this information.* | In this study, we place great emphasis on the participant consent process. For all users involved in filling out both paper-based and electronic questionnaires, the written consent approach was adopted. When distributing questionnaires, an informed consent form was provided concurrently. This form elucidated key information such as research objectives, methods, potential risks and benefits, data confidentiality measures, and participants' rights in an accessible language. After thorough reading and comprehension, participants signed in the designated area to confirm their consent to participate in the study. Additionally, in other information submitted to the system, we appended the English version of the informed consent form and a sample of the signed Chinese - version.  In the ethical statement within the “Methods” section of the paper and the online submission information, the type of consent obtained was clearly specified as written consent. To enhance the clarity and integrity of the paper, the following details were supplemented in the revised manuscript: In the ethical statement section, the main content framework of the informed consent form was described in detail, enabling readers to intuitively understand the key information. The processes to ensure a one-to-one correspondence between the informed consent form and the questionnaire during the collection of paper-based and electronic questionnaires were explained. For paper-based questionnaires, participants were required to sign the consent form prior to filling out the questionnaire. In the electronic questionnaire system, it was configured such that participants must read and agree to the informed consent form before they could enter the filling section. The specific content can be found in the red-marked revised parts corresponding to lines **651-656** in the revised manuscript. |
| *4. We note that you have indicated that there are restrictions to data sharing for this study. For studies involving human research participant data or other sensitive data, we encourage authors to share de-identified or anonymized data. However, when data cannot be publicly shared for ethical reasons, we allow authors to make their data sets available upon request. For information on unacceptable data access restrictions, please see http://journals.plos.org/plosone/s/data-availability#loc-unacceptable-data-access-restrictions.* | There are two main reasons for the data sharing restrictions in this study. On one hand, the research is currently in an in - depth expansion and analysis phase. It is necessary to conduct multi - dimensional cross - validations using the existing dataset to construct a more accurate research model. At this stage, the stability of the data is of utmost importance. Premature data disclosure is likely to lead to misinterpretation or abuse of the data, interfering with subsequent research and affecting the quality of the research outcomes. Therefore, it is not appropriate to disclose the data at this stage. On the other hand, the research findings of this study are innovative, and we are currently actively preparing for patent applications. During the patent application process, data confidentiality is crucial for the approval of the patent. Premature disclosure may expose the core content, trigger intellectual property disputes, and increase the risk of patent application failure. Thus, the data cannot be disclosed until the application procedures are completed. We understand the journal's requirements for data sharing and its intention to promote academic exchange. We have submitted the original survey data of this study in the system as “Supporting Information files.” Please refer to the attachment titled “*The original survey data of this study*.” Before the completion of this study, if researchers request access to the data for legitimate research purposes, we will strictly review their identities and intended use and provide the dataset through secure and compliant means. |
| *5. Please include your full ethics statement in the ‘Methods’ section of your manuscript file. In your statement, please include the full name of the IRB or ethics committee who approved or waived your study, as well as whether or not you obtained informed written or verbal consent. If consent was waived for your study, please include this information in your statement as well.* | We greatly appreciate your pointing out this issue. We have already supplemented a complete ethical statement in the “Methods” section of the paper as required. Our study has been approved by the Biomedical Ethics Committee of Hefei University of Technology, with the ethical approval number [HFUT20240620001H]. During the research process, for all participants, we adopted the method of written informed consent. We will elaborate in detail on the specific procedures for obtaining written informed consent. For example, when distributing questionnaires or conducting research activities, how to provide the informed consent form simultaneously, as well as the specific ways in which participants sign the consent form and the situation of record retention, etc. This is to ensure the integrity and clarity of the ethical statement and meet the publication requirements of your esteemed journal. The specific content can be found in the red-marked revised parts corresponding to lines **651-656** in the revised manuscript. |

II. ***Review Comments to the Author***

***Reviewer 1:***

1. ***The main issue with this study is its lack of originality. While the study’s focus on facial recognition payment in the field of elderly populations is meaningful, I believe it is somewhat insufficient for publication. Therefore, it is better to emphasize the originality in the Introduction section.***

Thank you for your insightful comments. In response to your concerns regarding the originality of this study, we have made substantial revisions to the Introduction section to better articulate its novelty. Specifically, we have integrated a dual-theoretical framework based on the Technology Acceptance Model (TAM) and User Experience Theory, underscoring its distinctiveness in examining the adoption of facial recognition payment (FRP) among older adults. Furthermore, we have elaborated on key factors related to elderly user characteristics and usage dimensions, including technology anxiety, technological self-efficacy, and perceived convenience, which are critical aspects that have received limited attention in existing research. To enhance clarity and conciseness, we have also refined the background section by streamlining redundant descriptions of demographic aging statistics and the commercialization of FRP, thereby ensuring a more focused discussion on the relationship between older users and FRP adoption. These revisions aim to strengthen the study’s originality and theoretical contributions. Please refer to the revised manuscript (lines **68–146**) for detailed modifications.

2. ***The study employs Technology Acceptance Model, but it is worth considering other theories in order to support the research model in the study.***

We sincerely appreciate the valuable comments provided by the reviewers. The Technology Acceptance Model (TAM) serves as the core theoretical framework of this study, playing a crucial role in exploring elderly users’ acceptance of facial recognition payment (FRP). However, we fully acknowledge that a single theoretical perspective may not comprehensively capture the multifaceted nature of this phenomenon. Therefore, building upon existing research, we have further integrated User Experience (UX) theory to establish a more comprehensive and robust theoretical foundation.Regarding variable selection, from the perspective of the Technology Acceptance Model, perceived convenience (PC) directly influences perceived ease of use (PEOU), while facilitating conditions (FC) provide external support, thereby impacting technology adoption. Additionally, technology anxiety (TA) and technology self-efficacy (TSE) play pivotal roles in shaping users’ confidence in and concerns about technology usage, collectively determining their acceptance intention. From the User Experience perspective, perceived enjoyment (PE) enhances users’ emotional engagement, while personal innovativeness (PI) affects their willingness to explore new technologies. Moreover, privacy risk (PR) is closely related to users’ sense of security and trust, ultimately influencing their adoption decisions. Based on these considerations, this study highlights the complementary relationship between the Technology Acceptance Model and User Experience theory within the theoretical framework, providing a more holistic explanation of elderly users' acceptance behavior toward facial recognition payment. In response to these refinements, this study has supplemented and revised the relevant literature in Chapter 2 (Literature Review) and made corresponding modifications across Chapters 3 to 7. Additionally, Figures 1 and 2 have been updated. All revisions have been marked in red in the relevant sections of the revised manuscript. Please refer to lines **200-259** in the revised manuscript and the corresponding modifications and additions in the subsequent chapters (marked in red).

3. ***It would be beneficial to add empirical studies for hypotheses 6 to 14***

Dear Reviewer, Thank you very much for your valuable comments. In response to the suggestion of “supplementing empirical research for Hypotheses 6 to 14”, we carefully designed a structured questionnaire and adopted a sampling method combining online and offline approaches to collect data from the elderly population aged 60 and above. We used SPSS 23.0 for preliminary analysis and employed AMOS 24.0 to conduct Structural Equation Modeling (SEM) analysis, and evaluated the model fit through multiple fit indices. The results show that Hypotheses 6 to 9, and 11 to 14 are supported or partially supported (p < 0.001, p < 0.05). For example, in Hypothesis 6, Perceived Convenience significantly and positively affects Perceived Usefulness (β = 0.853, p < 0.001), and in Hypothesis 14, Privacy Risk significantly and negatively affects Attitude (β = -0.148, p < 0.001), while Hypothesis 10 is not supported (p > 0.1). We have elaborated and presented the above-mentioned empirical research content in detail in **Chapter 4** (Research Methodology) and **Chapter 5** (Data Analysis and Results) of the paper.

4. ***Citing the following paper would help enhance the quality of the theoretical background in this study. Joo et al. (2024). Effects of foodservice consumers’ perceptions of face recognition payment on attitude, desire, and behavioral intentions: a cross-cultural study. Journal of Travel & Tourism Marketing, 41(3), 359-376. Hwang et al. (2024). Effects of motivated consumer innovativeness on facial recognition payment adoption in the restaurant industry: A cross-cultural study. International Journal of Hospitality Management, 117, 103646. Kim et al. (2024). The antecedents and consequences of task–technology fit of facial recognition payment systems in the restaurant industry: cultural differences. Journal of Hospitality and Tourism Technology, 15(3), 397-416. Hwang et al. (2024). An integrated model of artificially intelligent (AI) facial recognition technology adoption based on perceived risk theory and extended TPB: A comparative analysis of US and South Korea. Journal of Hospitality Marketing & Management, 33(8), 1071-1099.***

We sincerely appreciate your suggestion to enhance our study's theoretical background. We have incorporated the four recommended papers as you advised, with the citations located at lines **219-228** of the revised manuscript, marked as **32-34**. These papers, covering cross-cultural aspects, task - technology fit, and models based on perceived risk theory, enrich our theoretical foundation and are highly relevant to our research on elderly users’ acceptance of facial recognition payment. Thank you for your guidance that has significantly improved our work.

5. ***All measurement items should be presented.***

We sincerely appreciate your feedback regarding the presentation of all measurement items. In response, we have ensured that all relevant measurement items are comprehensively presented in **Table 3** of the manuscript. This table details the questionnaire items corresponding to each construct, including Perceived Ease of Use, Perceived Usefulness, Attitude, Behavioral Intention, Perceived Convenience, Perceived Enjoyment, Personal Innovation, Technological Self - Efficacy, Facilitating Conditions, Technology Anxiety, and Privacy Risk. Each item is accompanied by its specific details and relevant references, providing a clear and thorough view of how each variable was measured in our study. Furthermore, we have also made an explicit statement in the main text of the revised manuscript, specifically in lines **675-677**. This additional clarification further emphasizes our attention to detail and the comprehensiveness of our research approach. We believe this comprehensive presentation not only addresses your concern but also enhances the transparency and replicability of our research methodology. Thank you again for your valuable input, which has been instrumental in improving the quality of our work.

6. ***A discussion is also necessary for hypotheses that are not statistically significant.***

We are extremely grateful for your astute comment regarding the discussion of hypotheses that lack statistical significance. In response to your suggestion, we have thoroughly addressed the non-significant hypothesis in our study. Specifically, in lines **811-838** of the revised manuscript, we have dedicated substantial space to discussing the finding that Technological Self-Efficacy (TSE) did not significantly affect the technology use attitudes of older users, which contradicts our initial hypothesis. We first compared this unexpected result with previous research findings, highlighting the contrast with the majority of existing studies while also pointing out its consistency with some specific works like Mohammed et al.’s. Then, we delved into potential reasons for this non - significance. We proposed that the inherent simplicity of facial recognition payment technology, which simplifies the user interface and operational procedures through the “face scan to pay” mechanism, might be the key factor reducing the influence of TSE on user attitudes. We believe that this in-depth discussion not only meets your requirement but also enriches the understanding of our research results, demonstrating our comprehensive consideration of all aspects of the study. Thank you again for your invaluable feedback, which has been crucial in enhancing the quality and rigor of our research.

***7. The theoretical implications were presented in comparison with existing research, but they do not carry significant meaning.***

We sincerely appreciate your valuable comment regarding the theoretical implications of our study. In response to your concern, we would like to elaborate on the theoretical contributions we have made. In our research, we introduce a novel theoretical innovation by integrating the Technology Acceptance Model (TAM) and the User Experience Technology (UXT) framework to construct our research model. This integration is a significant departure from existing studies, as it allows us to comprehensively consider both the cognitive factors related to technology acceptance (from TAM) and the emotional and experiential aspects (from UXT) in the context of elderly users’ adoption of Facial Recognition Payment (FRP) systems. We have provided a detailed discussion of these theoretical contributions in Section 7 “Conclusion and Limitations” of the revised manuscript, specifically in lines **956-985**. Here, we not only explain how our integrated model enriches the understanding of the factors influencing elderly users’ technology acceptance but also highlight how it extends the application boundaries of traditional models. By incorporating variables such as Perceived Convenience, Technological Anxiety, Perceived Enjoyment, and Privacy Risk, we offer a more nuanced and comprehensive view of the technology acceptance process among the elderly. We believe that these theoretical advancements are not only relevant but also of substantial significance in the field of technology acceptance research, especially when focusing on specific user groups like the elderly. Thank you again for your feedback, which has encouraged us to further clarify and emphasize the importance of our theoretical contributions.

***Reviewer 2:***

1. ***This paper is well-written, and the ideas are presented in a clear manner.***

Thank you very much for your positive feedback on the quality of our paper and the clarity of our arguments. Your appreciation is truly encouraging, and we are committed to maintaining and further improving our research quality in future studies. Once again, we sincerely appreciate your support.

2. ***What is the reasoning behind reporting H2, H12, and H13 as ‘partially supported?’***

Thank you for your insightful comments on our study. The classification of H2, H12, and H13 as “partially supported” is based on the results of the path analysis conducted using Structural Equation Modeling (SEM) and follows established statistical significance thresholds, as outlined below:

p < 0.001: The hypothesis is classified as strongly supported, indicating a highly significant relationship.

0.01 ≤ p < 0.05: The hypothesis is categorized as partially supported, suggesting statistical significance, albeit with a weaker effect.

p > 0.05: The hypothesis is deemed not supported, indicating a lack of statistical significance.

According to this classification framework, the statistical significance levels of H2, H12, and H13 fall within the range of 0.01 ≤ p < 0.05, warranting their classification as “partially supported.” This categorization aligns with standard statistical interpretation practices in SEM-based hypothesis testing.

To enhance clarity, we have explicitly outlined the classification criteria in Section 5.2 and Table 7 of the manuscript, with a corresponding reference [161-165]. The specific details can be found in lines **739-744**, highlighted in red for easy reference.

3. ***What is a logical explanation for why TSE did not have a significant effect on attitude specifically in the context of elderly consumers?***

Thank you for your valuable feedback and for your insightful question regarding the absence of a significant effect of Technological Self-Efficacy (TSE) on attitudes toward Facial Recognition Payment (FRP) technology, particularly in the context of elderly consumers. In response to your question, we acknowledge that this finding may seem counterintuitive given the established literature. As discussed in our manuscript, we based our explanation on the observation that the “face scan to pay” mechanism of FRP significantly simplifies the user interface and operational procedures. This simplification reduces the reliance on self-efficacy in using the technology. Unlike traditional digital payment systems, which require multiple steps such as password input or QR code scanning, FRP operates in a highly intuitive and almost automatic manner, thereby enabling even individuals with lower confidence in their technological abilities to use it effortlessly.

Additionally, from the perspective of User Experience Theory (UXT), we have highlighted that elderly users tend to assess new technologies based on their perceived simplicity, convenience, and security rather than their technological competence. This insight aligns with the growing emphasis on age-friendly design, which aims to enhance technology adoption by minimizing cognitive and operational burdens. We have updated the manuscript to further emphasize the role of user experience and emotional design in shaping elderly users’ technology attitudes, as these factors play a crucial role in facilitating technology acceptance among older adults. We hope this explanation clearly clarifies the reasoning behind the results and addresses your concerns. We have incorporated these insights into the revised manuscript to provide a more comprehensive understanding of how technological self-efficacy interacts with other user experience factors to influence elderly users’ attitudes toward FRP. Please refer to lines **811-838** in the revised version for the detailed content.

4. ***The paper states that the data collection focused on individuals aged 60 and above, but Table 2 lists an age group from 55-60 years old. Were all participants over the age of 60 or were the female participants following the threshold of the Chinese official retirement age?***

Thank you for your insightful comment. We acknowledge the inconsistency in the original description and have clarified this in the revised manuscript. While our study primarily focuses on individuals aged 60 and above, we also included female participants aged 55-60 based on China’s official retirement age policy (55 for women and 60 for men). Given that retired individuals in this age group often exhibit technology adoption behaviors similar to those aged 60 and above, their inclusion allows for a more comprehensive analysis of elderly users’ attitudes toward FRP. To enhance clarity, we have explicitly clarified our sampling method in the revised manuscript (Lines **627-641**). Thank you again for your thorough review and valuable suggestions!

***5. Were all participants previously familiar with FRP systems or was there some type of informational materials given to the participants to help them gain an understanding of how FRP systems operate prior to completing the survey?***

Thank you for your valuable comments. To ensure that all participants in the survey have a certain level of understanding of the FRP system and can accurately respond to questions about their usage experience and attitudes, we have implemented strict criteria for participant selection. All participants must have prior exposure to the FRP system and possess a certain level of knowledge before completing the questionnaire. Additionally, to ensure participants fully understand the research content while filling out the questionnaire, we required all participants to sign an informed consent form before the survey. The informed consent form provided detailed information about the purpose, content, and requirements of the research.

To assist participants in filling out the questionnaire correctly, research assistants provided on-site explanations during the offline survey process. The informed consent form also included the contact details of the research assistants, allowing online participants to seek clarifications as needed. Therefore, we believe that participants’ understanding of the FRP system is adequate, which helps improve the validity and reliability of the data. To ensure transparency, we have submitted the English version of the informed consent form and a sample of the signed informed consent form from offline participants as supplementary materials in this revised submission for the reviewers’ reference. Additionally, we have supplemented the relevant content in the revised manuscript, with specific changes reflected in lines **633-636, 651-656.** We believe these measures will effectively minimize any potential misunderstandings participants may have when completing the questionnaire, thus ensuring the accuracy of the research data.

***Explanation of Other Modifications***

1. **Title Revision**

Based on the reviewers’ comments, we have rethought the theoretical innovation of the paper. By integrating the User Experience Theory (UXT) into the original TAM model, we have revised the title of the paper to: *Understanding Older Adults’ Adoption of Facial Recognition Payment: An Integrated Model of TAM and UXT.*

2. **Abstract Update**

In line with the overall revisions made to the paper, the abstract has also been updated accordingly.

3. **Section 3 and Table 1 Revision**

In accordance with the revisions made to the theoretical framework of the paper, the content of Section 3.1 has been updated. Additionally, **Table 1** has been revised to include three relevant studies on the integration of TAM and UXT in research, specifically references [30], [35], and [37], along with their associated details.

4. **Figure Revision**

In accordance with the adjustments made to the content of the paper, Figures 1 and 2 have been updated. The factors extended based on the TAM theory are represented in blue, while the factors extended based on the UXT theory are represented in orange.
